# Place vs. Response Learning: History, Controversy, and Neurobiology

**DOI:** 10.3389/fnbeh.2020.598570

**Published:** 2021-02-11

**Authors:** Jarid Goodman

**Affiliations:** Department of Psychology, Delaware State University, Dover, DE, United States

**Keywords:** place learning, response learning, hippocampus, dorsal striatum, memory systems, habit, spatial memory, stimulus-response learning

## Abstract

The present article provides a historical review of the place and response learning plus-maze tasks with a focus on the behavioral and neurobiological findings. The article begins by reviewing the conflict between Edward C. Tolman’s cognitive view and Clark L. Hull’s stimulus-response (S-R) view of learning and how the place and response learning plus-maze tasks were designed to resolve this debate. Cognitive learning theorists predicted that place learning would be acquired faster than response learning, indicating the dominance of cognitive learning, whereas S-R learning theorists predicted that response learning would be acquired faster, indicating the dominance of S-R learning. Here, the evidence is reviewed demonstrating that either place or response learning may be dominant in a given learning situation and that the relative dominance of place and response learning depends on various parametric factors (i.e., amount of training, visual aspects of the learning environment, emotional arousal, et cetera). Next, the neurobiology underlying place and response learning is reviewed, providing strong evidence for the existence of multiple memory systems in the mammalian brain. Research has indicated that place learning is principally mediated by the hippocampus, whereas response learning is mediated by the dorsolateral striatum. Other brain regions implicated in place and response learning are also discussed in this section, including the dorsomedial striatum, amygdala, and medial prefrontal cortex. An exhaustive review of the neurotransmitter systems underlying place and response learning is subsequently provided, indicating important roles for glutamate, dopamine, acetylcholine, cannabinoids, and estrogen. Closing remarks are made emphasizing the historical importance of the place and response learning tasks in resolving problems in learning theory, as well as for examining the behavioral and neurobiological mechanisms of multiple memory systems. How the place and response learning tasks may be employed in the future for examining extinction, neural circuits of memory, and human psychopathology is also briefly considered.

## Introduction

Learning theory in the first half of the 20th century was dominated by two broad opposing views regarding the mechanisms of animal learning and memory. According to the stimulus-response (S-R) view, animals acquire associations between stimuli (S) and responses (R) in the learning environment. For instance, in a maze learning situation, in which an animal learns to traverse a maze to retrieve food from a consistent goal location, visual or tactile stimuli in the learning environment may acquire the ability to activate a series of kinesthetic turning responses guiding behavior to the food reinforcer. In contrast, according to a competing cognitive approach termed purposive behaviorism, animals acquire meaningful relationships between stimuli in the learning environment, leading to the formation of “cognitive expectations.” These acquired expectations allow the animal to make inferences about what behavioral responses need to be made to retrieve the food reinforcer. Thus, according to the cognitive view, animals are not S-R automatons, but rather they make rational decisions based on expectation, reason, and purpose.

Noteworthy figureheads of the S-R view included Thorndike (1933), Guthrie (1935), and Spence (1936), and the investigator to provide perhaps the most complete iteration of the S-R view was Clark L. Hull in his profoundly popular book *Principles of Behavior* (Hull, 1943). On the other hand, the cognitive view of learning as described above was introduced by Tolman (1932) in another influential book titled *Purposive Behavior in Animals and Man*. Given the popularity of the views espoused by Hull and Tolman, the debate between S-R and cognitive learning theories transpired predominantly as a series of experiments comparing the Hullian vs. Tolmanian views of learning and memory.

As a means to resolve the debate, Tolman et al. (1946a) designed two plus-maze tasks^1^—a “response learning” task that requires the use of S-R learning and a “place learning” task that requires the use of cognitive learning. Soon after the introduction of Tolman’s place and response learning tasks, another laboratory designed a third version of the plus-maze, i.e., the dual-solution task, to examine the *relative use* of place and response learning (Blodgett and McCutchan, 1948). These three plus-maze tasks were widely adopted by other laboratories to assess the Hullian and Tolmanian approaches to learning and memory, amounting to the decade-long “place vs. response controversy” (Restle, 1957). As interest in the place vs. response controversy and the debate between cognitive and S-R learning theories waned, the use of these plus-maze tasks declined as well. However, this decline was only temporary, as the place and response learning tasks have re-emerged in recent years as important tools for investigating the behavioral and neurobiological mechanisms of different kinds of memory.

The present review provides a historical account of the place and response learning plus-maze tasks. The review begins by briefly describing the learning theories espoused by Tolman and Hull, as well as some of the historical factors that motivated their ideas. This is followed by a description of the place and response learning plus-maze tasks. The next section highlights the place vs. response controversy of the 1940s–1950s and how it was resolved by recognizing that various parametric factors determine whether a place or response learning dominates behavior. Next, the present review discusses some alternative methodologies for investigating place and response learning outside the classic Tolmanian plus-maze tasks. This is followed by an exhaustive review of the neurobiology of place and response learning, including the major brain structures and neurotransmitter systems that have been implicated. Finally, the present review considers the future of the place and response learning tasks, such as their potential use in research on neural memory circuits, extinction learning, revisions to memory systems theory, and human psychopathology.

## Tolman at the Choice-Point

**Figure 1:**
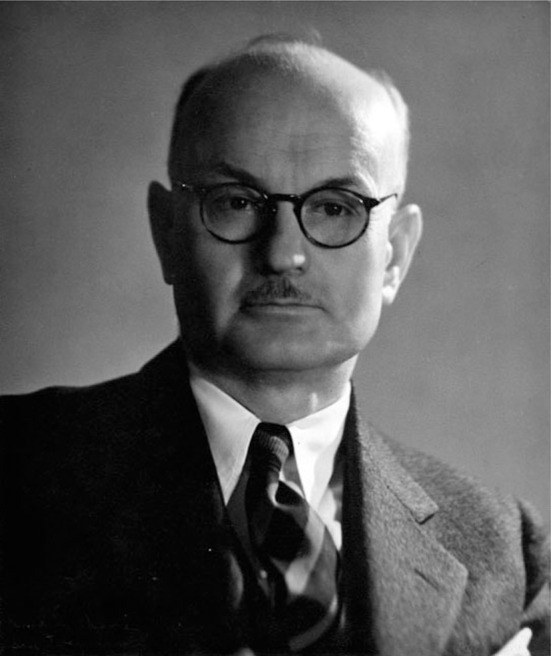
Edward Chace Tolman (1886‐1959).

In 1932, when Edward C. Tolman (Figure 1) published his book titled *Purposive Behavior in Animals and Man*, he became, at once, the father of purposive behaviorism and the figurehead leading the rebellion against the S-R view of learning. Not only were Tolman’s salvos evident in his writings, but he was also wont to criticize the theory in his less official correspondences. As one of his former students recounts:

…*in one lecture commenting on the limitations of S-R theory [Tolman] pointed out that, as there are important cognitive processes in the organism that intervene between the S and R, at the very least, an “O” (for “organism”) must be inserted. Moreover, as it is behavioral acts that occur and not muscle-twitch responses, the “R” should be changed to “B” for “behavior.” He then, with a sly grin (and no doubt with some residual New England guilt), referred to S-R theory as the “SOB” theory* (McGaugh, 2003, p. 19).

This amusing anecdote from one of Tolman’s students perhaps sheds light on the investigator’s distaste for the S-R view that in part motivated him to write *Purposive Behavior*.

Although Tolman became one of the most popular and energetic opponents of the S-R view, he did not always harbor a negative attitude toward the theory. As a graduate student at Harvard, Tolman took a course in comparative psychology, which was then taught by renowned psychologist Robert Yerkes. The textbook for the course was a seminal work by Yerkes’ friend and colleague, John B. Watson, titled *Behavior: An Introduction to Comparative Psychology*. Tolman at the time regarded Watson’s behaviorism as a “tremendous stimulus and relief” from the alternative introspective approach being employed by other psychologists around this time (Tolman, 1952, p. 326). However, Tolman also had some reservations about Watsonian behaviorism:

*I… did not like Watson’s over-simplified notions of stimulus and response. Nor did I like his treatment of each single stimulus and each single response as a quite insulated phenomenon that has practically no relation to any other stimuli or any other responses. That is, I was already becoming influenced by Gestalt psychology and conceived that a rat running a maze must be learning a lay-out or pattern and not just having connections between atom-like stimuli and atom-like responses “stamped in” or “stamped out,” whether by exercise or by effect* (Tolman, 1952, p. 329).

Thus, it may have been a lack of confidence in Watson’s overly reductionist view of behavior that led Tolman to begin considering the alternative Gestalt views of learning that had been emanating from Germany and gaining popularity in the US. It is tempting to imagine Tolman during these formative years as a rat at the critical intersection—or choice point—of a maze, in which he had the option to turn one way and continue in the spirit of Watsonian behaviorism or turn the opposite way and subscribe wholeheartedly to Gestalt psychology. However, it would turn out that Tolman took neither of these paths. Instead, he charted a new path toward his own view of behavior—a theory that combined Watsonian behaviorism with select principles of Gestalt psychology.

In *Purposive Behavior*, Tolman suggested that behavior is motivated by purpose, cognition, and expectation. That is, animals acquire meaningful relationships between stimulus objects in the environment, to the extent that an animal learns that interacting with one particular object a certain way (e.g., turning right at the intersection) will lead to the opportunity to interact with another object (e.g., turning left at the next intersection) and so forth. Knowledge about how stimuli in the environment are related to each other is encoded through stimulus-stimulus (S-S) associations. All relevant stimuli in the environment and the meaningful associations between them are combined and encapsulated in what Tolman called a “sign-gestalt expectation” or, on a larger scale, a “field expectation.” Stimuli in the environment may activate the whole sign-gestalt or field expectation, which can then be employed by the animal to purposefully guide behavior toward the pleasurable state of affairs (e.g., food, avoidance of threat, et cetera). Tolman’s ideas relating to sign-gestalt and field expectations were later expounded upon in his seminal article on cognitive maps (Tolman, 1948). In this article, Tolman suggested that animals (including people) acquire a variety of cognitive maps—including not only allocentric maps of space, but also more abstract interpersonal maps—that contribute to behavior, thought, and (on a speculative note) psychopathology (Tolman, 1948).

It is important to emphasize that although Tolman’s inclination toward a cognitive view of learning was partially motivated by a skepticism surrounding strict Watsonian behaviorism, his cognitive views were corroborated through extensive behavioral research, much of which was conducted in his laboratory at Berkeley. Tolman used a variety of mazes to show that animals can acquire cognitive maps of a learning environment, and they could use these maps to guide running behavior toward a palatable food reinforcer. For instance, contrary to the S-R view of learning, animals could make inferences about there being shortcuts in the maze and generate a novel series of navigational responses based on those inferences (Tolman et al., 1946b). The experiments coming from Tolman’s laboratory promoted a shift in the field from a rather spartan S-R view toward a more purposeful, cognitive view of behavior. However, just as Tolman’s cognitive expectancy theory was gaining traction in the field, another investigator took the stage and touted an impressive rejuvenation of the S-R view that could not be easily ignored.

## A Hull in the Machine

**Figure 2:**
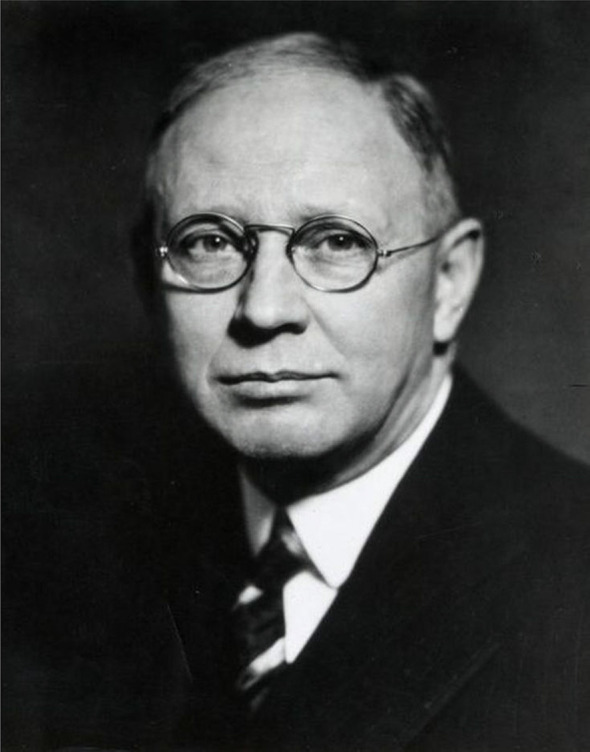
Clark Leonard Hull (1884‐1952).

**Figure 3:**
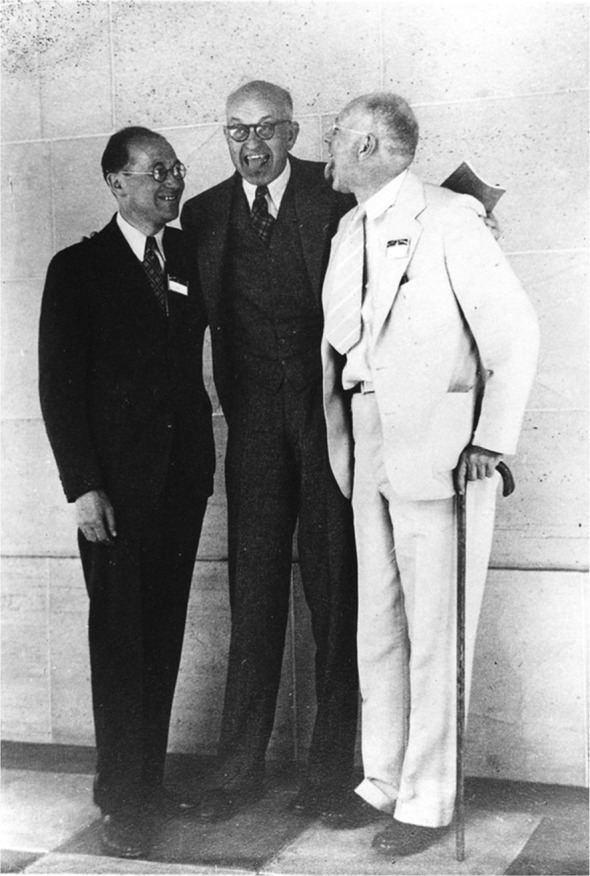
From left to right: Kurt Lewin, Edward Tolman, and Clark Hull at the 47th Annual Meeting of the American Psychological Association in 1939. Tolman and Hull stick their tongues out at each other as a playful expression of their opposing views on learning (from the Geoffrey W. H. Leytham collection, Archives of the History of American Psychology, the Drs Nicholas and Dorothy Cummings Center for the History of Psychology, University of Akron).

When Clark L. Hull (Figures 2, 3) began studying psychology, he—like Tolman—developed a fascination with Watsonian behaviorism. However, also like Tolman, he had some reservations about the theory. Hull disagreed with some of Watson’s “dogmatic claims,” and the result of his disagreement “was a belated conversion to a kind of neo-behaviorism—a behaviorism concerned with the determination of the quantitative laws of behavior and their deductive systemization” (Hull, 1952, p. 154). Drawing from his love of mathematics and his professional expertise in chemistry and engineering, Hull developed an intricate series of “mathematico-deductive” formulas to explain and predict observable behavior (Hull et al., 1940). These formulas were leaps and bounds above the primitive S-R associations being proposed by classical behaviorists (Thorndike, 1898; Watson, 1914).

Although Hull and Tolman were similar in their urge to break away from the restrictions of Watsonian behaviorism, Hull, unlike Tolman, refrained from incorporating in his theory what he viewed as teleological concepts, such as purpose and expectation. He believed that such concepts were the unfortunate byproducts of anthropomorphic subjectivism (i.e., the tendency to regard animals as having human thoughts and feelings). To safeguard oneself against these pitfalls, Hull suggested that we regard

*the behaving organism as a completely self-maintaining robot, constructed of materials as unlike ourselves as may be. In doing this it is not necessary to attempt the solution of the detailed engineering problems connected with the design of such a creature. It is a wholesome and revealing exercise, however, to consider the various problems in behavior dynamics which must be solved in the design of a truly self-maintaining robot* (Hull, 1943, p. 27).

Hull’s inclination to view organisms as automatons that operate without purpose and free will remains evident in his mathematico-deductive view of behavior.

According to Hull, the probability of a particular behavior being performed (i.e., reaction potential) was a function of drive and habit strength. Habit strength, he defined, as the degree to which a stimulus (S) has the capacity to activate a response (R), with the performance of R leading to drive reduction. For instance, if a hungry rat hears a tone (S) and then presses a lever (R) resulting in the delivery of a favorable food outcome (O), consumption of the food will lead to drive reduction (i.e., less hunger), and thus the S-R association between the tone and the lever-press will be strengthened. Habit strength increases throughout many iterations of S being paired with R. Over time, the S can activate the R automatically, even under conditions of low drive. Thus, much of the learned behavior according to Hull is a series of S-R habits.

Hull’s theory, though impressive in its completeness and explanatory power, was harshly criticized by cognitive learning theorists, including Tolman and his colleagues (e.g., Tolman, 1948; Gleitman et al., 1954). Soon after the publication of Hull’s neobehaviorist manifesto titled *Principles of Behavior*, Tolman’s laboratory developed a new paradigm that placed the Hullian “habit” and Tolmanian “cognitive” theories in competition with each other. This Tolmanian paradigm was adopted by other laboratories and served as the battleground for the debate between S-R and cognitive views of learning for many subsequent and contentious years.

## Tolman vs. Hull: Development of the Place and Response Learning Plus-Maze Tasks

Early on, it became clear to investigators that if an animal is placed in a maze with food consistently placed at another “goal end” of the maze, the animal will eventually learn to retrieve the food (Small, 1901). However, exactly *how* animals learned to find the food or *what* animals acquired that enabled them to guide behavior to the rewarded location remained debatable. According to Tolman and colleagues, there were three potential explanations worth considering:

1. Such training may have produced a disposition in the rats to run on a path that has certain specific characteristics (e.g., knotholes of such and such a pattern, or the like) and to avoid running on all paths which have certain other specific characteristics.
2. Such training may have produced a disposition to turn right whenever they come to the choice point.
3. Finally, such training may have produced a disposition to orient towards the place where the food is located (e.g., under the window, to the left of the radiator, et cetera; Tolman et al., 1946a, p. 221).

Tolman and his colleagues quickly ruled out the first explanation based on earlier findings from Honzik (1936), suggesting that it was difficult for rats to use intramaze cues to guide behavior. However, they suggested that no studies as yet had directly compared the last two explanations. Is it the case that animals acquire a response (i.e., consistent with the Hullian S-R view of learning), or do animals learn to go to a place (i.e., consistent with Tolman’s cognitive view of learning)? Tolman et al. (1946a) developed two plus-maze tasks to examine these hypotheses.

### The Place Learning Task and the Response Learning Task

**Figure 4:**
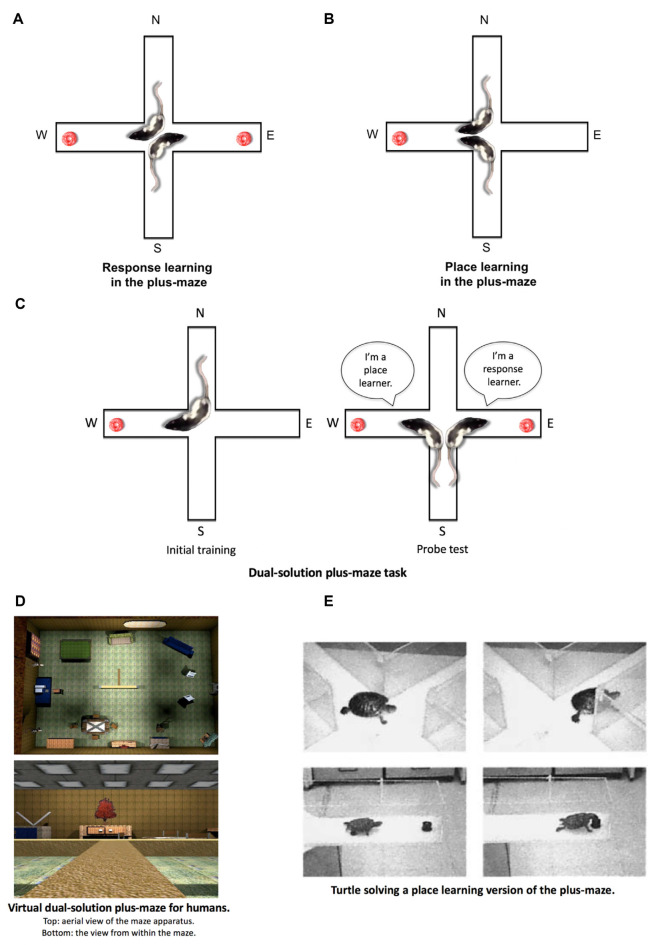
Place and response learning in the plus-maze.** (A)** In the response learning plus-maze task, a rat is released from opposite start arms (e.g., N and S) throughout training, and the same body-turn response at the choice point is reinforced. For example, when the rat is released from the N arm, food is located in the W arm. When the rat is released from the S arm, food is located in the E arm. In this example, a right-body turn at the choice point is being reinforced. **(B)** In the place learning plus-maze task, a rat is released from opposite start arms throughout training, while the food remains in a consistently reinforced spatial location.** (C)** During initial training in the dual-solution plus-maze task, a rat is released from a consistent start arm (e.g., N) with food also located in a consistent spatial location (e.g., W). A rat can learn to retrieve food using a place learning strategy (i.e., go to the same place) or a response learning strategy (i.e., make the same body turn at the choice point). To determine how the rat learned to solve the task, a probe trial is conducted in which the rat is started from the opposite arm (e.g., S). If the rat continues to make the same body-turn at the choice point, the rat is labeled a response learner. If the rat makes the opposite body-turn to return to the reinforced spatial location, the rat is labeled a place learner. **(D)** A virtual version of the dual-solution plus-maze task may be employed to study place and response learning in human subjects (images from Astur et al., 2016). **(E)** Place and response learning tasks have been employed to study learning and memory across a variety of species aside from rodents and humans, including turtles, salamanders, chickens, horses, and sharks, among others (image from Salas et al., 2003 with permission from S. Karger AG, Basel).

Tolman’s laboratory used a plus-maze that consisted of four arms arranged in a cross (+) formation. Two opposite arms (e.g., North and South) were designated as start arms from which the animals were released during maze training, and the other two arms (e.g., East and West) were designated as goal arms that may contain food reward during training. The two tasks that Tolman et al. (1946a) had run in the plus-maze were called the “place learning” and “response learning” tasks. In the place learning task (see Figure 4B), animals were released from the opposite starting positions, and a palatable food reward was located in a consistent goal arm. Thus, animals presumably needed to learn the spatial location of the food reward to accurately guide behavior from different starting positions to the rewarded spatial location. In the response learning task (Figure 4A), animals were also released from opposite starting positions, but the food reward in this case was rotated to opposite goal arms in such a way that for animals to quickly retrieve the food, they needed to make a consistent body-turn response. For instance, if rats were released from the North arm, the food reward was in the West arm. If the animal were released from the South arm, the food reward was in the East arm. Thus regardless of where a rat was released from, the rat needed to learn a consistent right body-turn to quickly retrieve the food.

Tolman et al. (1946a) wanted to determine which task animals learned faster. It was assumed at this time that if animals learned one task faster than the other, then the type of learning underlying the former task is more dominant or “natural” to the animals than the type of learning underlying the other, more slowly acquired task. The findings of their study indicated that animals learned the place learning task much faster than the response learning task, and therefore the investigators concluded that “both kinds of dispositions may be acquired by the rat, but that the disposition to orient towards the goal is simpler and more primitive than the disposition to make right turns” (Tolman et al., 1946a, p. 228). However, many subsequent plus-maze experiments challenged Tolman’s conclusion.

### The Dual-Solution Plus-Maze Task

Another team of investigators who were critical of Tolman’s findings provided an alternative version of the plus-maze task (commonly referred to as the dual-solution plus-maze) to examine the relative use of place and response learning. Hugh C. Blodgett, who obtained his Ph.D. under the guidance of Tolman at Berkeley, designed this dual-solution plus-maze task in his laboratory at the University of Texas (Blodgett and McCutchan, 1948). Over the course of maze training in the dual-solution plus-maze task, rats were released from a consistent starting position in the plus-maze (e.g., North), and food was placed in a consistent goal arm (e.g., West). This maze task was conducted in a homogenous visual environment with a single spatial cue (i.e., a 10 inch reflective disc) placed directly outside the maze. During the initial acquisition phase of the task, animals could find the food by acquiring either a place learning strategy (i.e., using the disc as a spatial cue to guide behavior to the food location) or a response learning strategy (i.e., making a consistent body-turn response at the choice-point). In either case, behavior looked the same during initial training. To determine which strategy the animals employed, a probe trial was conducted in which the reflective disc was moved to the opposite side of the room. Rats that had been using the disc as a spatial cue to find the food location during initial training (i.e., a place strategy) would end up going to the opposite maze arm during this probe trial. In contrast, rats that had learned to make a consistent body-turn during initial training (i.e., a response strategy) would continue to do so during the probe trial, while disregarding the change in the extra-maze spatial environment.

A surprising result from this original dual-solution plus-maze study from Blodgett and McCutchan (1948) was that during the probe trial, rats predominantly made the same turning response. These findings suggested that animals preferentially acquired and/or expressed a response learning strategy instead of a place learning strategy. The authors concluded that under their experimental conditions and in contrast to what Tolman et al. (1946a) previously suggested, “a response disposition is… stronger than a place disposition” (Blodgett and McCutchan, 1948, p. 23).

It should be noted that the dual-solution plus-maze task has evolved since its original iteration from Blodgett and McCutchan (1948). One of the problems with their version of the task was the use of a single spatial cue. Even though rats using the spatial cue were labeled as place learners in their study, using a single spatial cue to guide behavior in a maze situation may also be viewed as a form of S-R response learning to the extent that an animal may learn to simply run toward (R) the extra-maze cue (S). To provide an experimental maze situation conducive to place learning, an array of extra-maze spatial objects must be made available, allowing the subject to build a spatial cognitive map of the environment to guide behavior (Tolman, 1948). To this aim, later versions of the dual-solution plus-maze task have employed heterogeneous visual environments that contain multiple extra-maze objects. The initial training phase of the task has remained the same; however, the probe trial has been modified to preclude the moving of spatial objects around the room. Instead, during the probe trial, the rat is begun from the arm opposite to the original start arm (e.g., Packard and McGaugh, 1996). For example (see Figure 4C), if during initial training the rat is consistently placed in the North arm with food in the West arm, the rat begins the probe trial from the South arm. During the probe trial, if the rat continues to make the same right body-turn response at the choice point (i.e., running away from the original food location on the West arm), they are labeled as a “response-learner.” If instead, the rat makes a left turn at the choice point (i.e., running toward the original food location on the West arm), they are labeled as a “place-learner.”

## The Place vs. Response Controversy

Immediately following the original plus-maze experiments conducted by Tolman and Blodgett, other experimenters began using the same plus-maze tasks to examine cognitive vs. S-R views of learning. Proponents of Tolman’s cognitive view of learning believed that place learning was more dominant or natural to the animal than response learning, while proponents of Hull’s S-R view believed the opposite. This conflict set the stage for the place vs. response controversy (Restle, 1957)—that is, the debate over whether animals in the plus-maze are naturally place-learners or response-learners—and directly motivated the immediate widespread use of the place and response learning plus-maze tasks in the 1940s–1950s. However, similar to the original studies conducted in the laboratories of Tolman and Blodgett, these experiments yielded mixed findings. In some cases, investigators found place learning to be dominant, whereas in other cases response learning was dominant. The eventual resolution to this conundrum was that either place or response learning could be dominant in these plus-maze tasks and that whether a particular kind of learning was dominant depended on a host of parametric factors (for reviews, see Restle, 1957; Packard and Goodman, 2013).

The role of parametric factors on the relative use of different learning strategies was perhaps originally envisaged by Tolman. In his famous article on cognitive maps, Tolman (1948) suggested that rats and humans alike acquire cognitive maps to guide our thoughts and behavior and that cognitive maps vary in size and detail. Tolman believed that large and detailed cognitive maps allow for animals to flexibly generate new routes from novel starting positions and to take shortcuts when shorter paths are suddenly made available. On the other hand, a relatively slim “strip-map” that is lacking in detail may allow the animal to undertake a simple navigational response from point A to point B, but would not allow for animals to take shortcuts or to quickly reach the goal location from a novel starting position. Thus, broad and comprehensive maps may allow for place learning, whereas narrow-strip maps may allow for response learning.

Tolman suggested that the relative smallness or bigness of a particular cognitive map may be influenced by a variety of factors. Tolman writes:

…*what are the conditions which favor narrow strip-maps and what are those which tend to favor broad comprehensive maps? There is considerable evidence scattered throughout the literature bearing on this question both for rats and for men. Some of this evidence was obtained in Berkeley and some of it elsewhere… I can merely summarize it by saying that narrow strip-maps rather than broad comprehensive maps seem to be induced: (1) by a damaged brain; (2) by an inadequate array of environmentally presented cues; (3) by an overdose of repetitions on the original trained-on path; and (4) by the presence of too strongly motivational or of too strongly frustrating conditions* (Tolman, 1948, p. 206–207).

While this excerpt pertains directly to the factors influencing the relative narrowness or comprehensiveness of a cognitive map, it also serves as an impressive list of the major factors influencing the relative dominance of place learning or response learning. The present section provides an overview of the behavioral factors influencing place and response learning, many of which were described above by Tolman, such as the amount of training, the visual aspects of the learning environment, and the emotional state of the organism. It should be noted that although many of these factors were first identified during the place vs. response controversy of the 1940s–1950s, this section also includes modern research to provide a more comprehensive discussion of the prominent factors influencing place and response learning.

### Factors Influencing Place and Response Learning

#### Amount of Training

According to the Hullian view of learning, the strength of an S-R habit is partially a function of the number of times that the S has been paired with the R. Therefore, it is reasonable to predict that after limited training an S-R association may be weak, allowing for other learning mechanisms to guide behavior. This prediction is consistent with the findings from studies using single-solution versions of the place and response learning tasks. That is, as previously mentioned, more trials are required for animals to learn the S-R response learning version of the plus-maze relative to the cognitive place learning version of the task (Tolman et al., 1947). Moreover, in the dual-solution task, when animals are given a probe trial after limited training, most animals demonstrate a place learning strategy, whereas, after extensive training, animals predominantly display response learning on the probe trial (Ritchie et al., 1950; Hicks, 1964; Packard and McGaugh, 1996; Packard, 1999). Interestingly, a more recent study demonstrated that the shift to response learning may be blocked if the animal is prompted to perform a concurrent working memory problem throughout task acquisition (Gardner et al., 2013). Also, in contrast to the “dry” appetitive dual-solution maze tasks, rats in water maze versions of the place and response tasks do not always show an initial preference for a place learning strategy. Research suggests that rats often display no preference (McDonald and White, 1994) or they show an initial preference for response learning and then shift to place learning after further training (Asem and Holland, 2013, 2015; Farina and Commins, 2020; Gasser et al., 2020). The initial expression of response learning in the water plus-maze may be partially explained by swimming stress (see “Emotional Arousal” section below).

#### Massed vs. Spaced Training

Evidence indicates that place and response learning may also differentially benefit from massed and distributed practice. During massed training, trials are separated by short inter-trial intervals, whereas during distributed (or spaced) training, trials are separated by considerably longer inter-trial intervals. Place learning is acquired quickly when using a massed-training protocol, in which trials are separated by 30 s, and slowly when using a distributed protocol, in which trials are separated by 15–30 min (Thompson and Thompson, 1949; Wingard et al., 2015). In contrast, response learning is acquired more slowly when using the massed-training protocol and is relatively faster when using the distributed protocol (Thompson and Thompson, 1949; Wingard et al., 2015).

#### Visual Aspects of the Learning Environment

Place and response learning in the plus-maze may also be influenced by the visual learning environment. In learning environments containing abundant extra-maze visual stimuli (termed heterogeneous visual surrounds), place learning is acquired faster than response learning, and a place learning strategy is preferred over a response learning strategy in dual-solution versions of the task (Tolman et al., 1946a, 1947; Blodgett and McCutchan, 1948; Blodgett et al., 1949; Tolman and Gleitman, 1949; Galanter and Shaw, 1954; Waddell et al., 1955). Visually heterogeneous learning environments may favor place learning by allowing animals to acquire a cognitive spatial map (Tolman, 1948). In contrast, learning environments containing few or no extra-maze visual cues (i.e., homogenous visual surrounds) allow for response learning to be acquired faster than place learning and lead to the use of response learning strategies over place learning strategies in the dual-solution plus-maze (Blodgett and McCutchan, 1948; Ritchie et al., 1950; McCutchan et al., 1951; Hill and Thune, 1952; Scharlock, 1955). Interestingly, the addition of extra-maze visual cues, which makes the learning environment more heterogeneous, impairs acquisition in a response learning task (Chang and Gold, 2004). It is possible that extra-maze cues stimulate the acquisition of a cognitive map and that animals using this cognitive map of the learning environment are more likely to go to the same place where they found food on a previous trial. This would lead to errors in a response learning task where the reinforcer is shifted to different spatial locations but would allow accurate performance in a place learning task where the reinforcer remains in a consistent spatial location. On the other hand, a relatively homogenous visual surround would presumably prevent the acquisition of a spatial cognitive map, which could: (1) lead to faster acquisition of response learning by eliminating spatial interference; and (2) impair place learning by preventing the animal from encoding the spatial location of the reinforcer.

#### Emotional Arousal

Another factor that profoundly influences place and response learning is stress and anxiety (for review, see Packard and Goodman, 2012; Goodman et al., 2017a; Packard et al., 2018). Behavioral stressors, such as restraint stress or exposure to predator odor, enhance acquisition in the response learning plus-maze task and lead to greater use of response learning over place learning in the dual-solution plus-maze task (Sadowski et al., 2009; Leong and Packard, 2014; Taylor et al., 2014). Chronic restraint and unpredictable shock also lead to greater response learning in other kinds of maze tasks (Kim et al., 2001; Schwabe et al., 2008). Aside from behavioral stressors, high levels of trait anxiety or hypertension favor response learning in the plus-maze and in a dual-solution version of the Morris water maze (Robertson et al., 2008; Wells et al., 2010; Hawley et al., 2011). Finally, conditioned emotional stimuli (e.g., a tone previously paired with a shock) may also enhance response learning and lead to greater use of response learning strategies over place learning strategies in the plus-maze (Leong et al., 2015; Goode et al., 2016). Presentation of a conditioned emotional stimulus may similarly promote the use of a response learning strategy in a dual-solution Morris water maze (Hawley et al., 2013).

Systemic infusions of stress hormones (e.g., corticosterone or epinephrine) or anxiogenic drugs (e.g., α-2 adrenoreceptor antagonists yohimbine or RS 79948-197) appear to mimic the effects of trait anxiety and behavioral stressors by enhancing response learning (Packard and Wingard, 2004; Elliott and Packard, 2008; Wingard and Packard, 2008; Packard and Gabriele, 2009; Leong et al., 2012). The enhancing effect of corticosterone or RS 79948-197 on response learning may be blocked by concurrent infusion of anxiolytic drugs (Leong et al., 2012; Goodman et al., 2015). Importantly, the enhancement of response learning following stress/anxiety has not only been demonstrated in rats using plus-maze tasks (Packard and Wingard, 2004) but has also been observed using response learning or “habit” memory tasks designed for human subjects (Schwabe et al., 2007, 2008, 2010, 2013; Schwabe and Wolf, 2009, 2010; Guenzel et al., 2014; Goldfarb et al., 2017; Goodman et al., 2020; Zerbes et al., 2020).

The enhancement of response learning may be attributed to the impairing effect of stress/anxiety on spatial memory processing. Infusions of anxiogenic drugs impair acquisition in a place learning version of the plus-maze, and similar doses enhance acquisition of response learning (Wingard and Packard, 2008; Packard and Gabriele, 2009; Sadowski et al., 2009). Consistent with the idea that the memory systems mediating place and response learning compete with each other in some learning situations (Poldrack and Packard, 2003), stress/anxiety may enhance response learning and lead to greater use of response learning strategies indirectly by impairing the function of the memory system mediating place learning.

#### Biological Sex

The potential influence of biological sex on place and response learning has also received some investigation. As reviewed previously, rats typically prefer a place learning strategy in the early stages of training in the dual-solution plus-maze and gradually shift toward a response learning strategy following extensive additional training; however, recent evidence indicates that only male rats display this shift in preference, whereas the strategy preference of female rats depends on estrogen levels (for reviews, see Korol, 2004, 2019). Female rats at proestrus (i.e., when estrogen levels are high) predominantly display place learning, whereas female rats at estrus (i.e., when estrogen levels are relatively low) predominantly display response learning (Korol et al., 2004; see also Korol and Kolo, 2002; Zurkovsky et al., 2006, 2007; Zurkovsky et al., 2011). In contrast, other research has found no preference between spatial and stimulus-response strategies in female rats even when the estrous cycle is taken into account, relative to male rats which continue to show either a place or response learning preference under various conditions (Grissom et al., 2012, 2013; Hawley et al., 2012). Also, men and women do not differ significantly from each other in terms of strategy preference across a variety of dual-solution tasks (e.g., Iaria et al., 2003; Schwabe et al., 2007, 2008; Andersen et al., 2012; Schwabe and Wolf, 2012).

### The End of the Place vs. Response Controversy

The general observation that the relative dominance of place and response learning depended on a myriad of experimental variables provided a potential resolution to the place vs. response controversy of the 1940–50s. In a highly cited review article summing up the findings from this era, Frank Restle (a prominent cognitive psychologist at the time) had this to say:

*There is nothing in the nature of a rat which makes it a “place” learner or a “response” learner. A rat in a maze will use all relevant cues, and the importance of any class of cues depends on the amount of relevant stimulation provided as well as the sensory capacities of the animal… The writer’s general conclusion is that further “definitive” studies of the place-vs.-response controversy, to prove that rats are by nature either place or response learners, would be fruitless*… (Restle, 1957, p. 226–227).

It is reasonable to infer that Restle’s general conclusion that further analysis of the place vs. response question would be “fruitless” was probably espoused by his contemporaries. A comment on Restle’s review, which was published about a decade later, noted that “since the appearance of Restle’s article, few further studies have been published dealing with the place vs. response issue” and that “it is unlikely that the issue will ever be reopened in its earlier form” (Goldstein et al., 1965, p. 229). In hindsight, we can determine that this prediction was correct—investigators were no longer concerned about which kind of learning was more “natural” to the animal. However, these authors had failed to foresee just how valuable the place and response learning tasks would become when later investigators began to examine the neural substrates of learning and memory.

It should be emphasized that research on the neurobiology of place and response learning took place not only in the plus-maze but in a variety of other maze tasks. Thus, before discussing the neurobiology of these different kinds of learning, it will be important to briefly review such alternative approaches to studying place and response learning.

## Alternative Place and Response Learning Tasks

The plus-maze tasks originating from the laboratories of Tolman and Blodgett—that is, the place learning task, response learning task, and dual-solution task—attracted the attention of numerous other investigators who later employed the tasks to examine Hullian S-R and Tolmanian cognitive views of learning. However, not all investigators replicated the original designs; some continued to modify them or designed completely new tasks to examine place and response learning (e.g., virtual versions of the dual-solution plus-maze; Figure 4D). These novel tasks allowed investigators to examine other aspects of place and response learning, while also providing certain advantages over the original designs. Below is a list of some of the alternative methods for studying place and response learning.

### Water Plus-Maze Tasks

Place and response learning tasks, including the dual-solution task, may be readily conducted in a water plus-maze (e.g., Schroeder et al., 2002; Packard and Wingard, 2004; Wingard and Packard, 2008). These tasks are conducted in a manner identical to the appetitive, food-reinforced versions described above. However, instead of rats running to retrieve food rewards, rats are placed in a plus-maze filled with water and must swim to an invisible escape platform hidden in one of the goal arms. Thus, in contrast to the appetitive versions of the place and response learning tasks, which involve positive reinforcement (i.e., food reward), the water plus-maze involves negative reinforcement (i.e., mounting an invisible platform to escape the water). The water plus-maze provides some advantages over the original appetitively reinforced tasks. Whereas animals trained in the appetitive versions need to be food-restricted for several days to motivate food foraging behavior, animals trained in the water maze do not need any prior food deprivation. Not having to deprive laboratory animals of food not only saves time, but it also rules out potential confounding variables, such as the influence of hunger and food deprivation on learning and memory. Also, animals tend to learn the water maze versions of the task much quicker than the appetitive versions, allowing investigators to complete experiments faster and increase experimental throughput. Finally, the water plus-maze tasks appear to depend on the same neurobiological systems as the appetitive tasks (Schroeder et al., 2002; Compton, 2004; Asem and Holland, 2015), suggesting that the underlying learning mechanisms may also be the same. However, as noted earlier, water maze tasks differ from the original appetitive “dry” maze tasks, in that emotional arousal induced by swimming stress potentially modulates memory formation and strategy preference in the water maze (see “Emotional Arousal” section above).

### Radial Arm Maze

**Figure 5:**
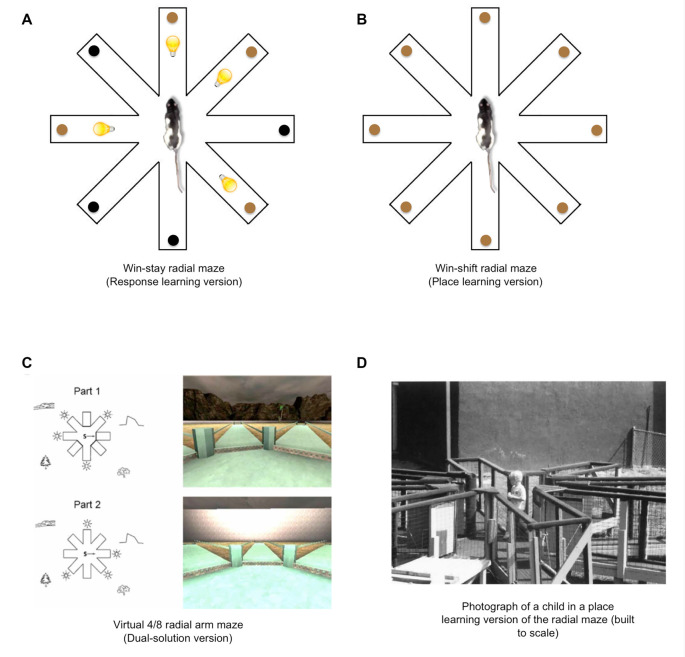
Place and response learning in the eight-arm radial maze.** (A)** In the “win-stay” radial maze task, light cues signal whether food is available at the end of each arm. This task promotes S-R response learning to the extent that the light cues (S) become associated with approach behavior (R). **(B)** In the win-shift radial maze, a rat must employ extra-maze spatial cues to locate food and avoid re-entries into arms in which food was already retrieved, thus promoting the use of place learning. **(C)** A virtual 4/8 dual-solution version of the radial maze may be employed to examine the relative use of place and response learning in human subjects. Each trial of this task is divided into two parts (Part 1 and Part 2). During Part 1, four of the eight arms are blocked with a wall, and the participant is instructed to enter the four open arms and retrieve hidden reward objects. During Part 2, all of the arms are open, and the participant is instructed to retrieve the remaining reward objects from the arms that were previously blocked. To avoid arm re-entries, the participants may employ a place learning strategy (i.e., refer to extra-maze spatial cues to guide behavior to the correct arms) or a response learning strategy (i.e., memorizing a series of egocentric responses leading to the correct arms). During a subsequent probe trial, the extra-maze spatial cues are blocked from view, preventing the use of a place strategy. Therefore, more errors during this probe trial suggest the use of a place strategy, whereas fewer errors suggest the use of a response strategy (images from Bohbot et al., 2012). **(D)** A 4-year-old child searches for rewards in a place learning version of the eight-arm radial maze (image from Overman et al., 1996).

In the radial arm maze, place and response learning can be assessed through “win-stay” and “win-shift” versions of the maze. In the S-R response learning or “win-stay” radial maze (Packard et al., 1989; Figure 5A), four of the eight arms in a radial maze are reinforced and signaled with a light stimulus, and rats may go to each of the illuminated arms twice within a daily training session to retrieve food. In this task, animals presumably acquire an S-R association between the light stimulus (S) and the approach response (R), whereas entries into the unlit arms are scored as errors. On the other hand, in the place learning or “win-shift” version of the radial maze (Figure 5B), rats may visit each of the eight arms once within a daily training session to retrieve food reward, whereas re-entries into previously visited arms are scored as errors. Importantly, arms containing food are not marked with any proximal cues, and therefore the animal must presumably rely on allocentric spatial cues to determine which arms were already visited and which arms still contain food. It should be noted that, while studies using the radial maze have shown evidence for distinct roles of the hippocampal and striatal neural systems in learning and memory, the radial maze has also been useful in showing how these two systems can interact in a cooperative manner (McDonald and White, 1995).

Interestingly, these radial maze tasks, although primarily conducted with rodents, have also been adapted to examine place and response learning in humans. These tasks typically involve computer-generated maze environments that the subject can navigate using a keyboard or joystick (see Figure 5D; e.g., Bohbot et al., 2004, 2007; Banner et al., 2011; Horga et al., 2015; Hussain et al., 2016b; Goodman et al., 2020). However, some investigators have examined “place and response-learning” using a built-to-scale radial arm maze that human participants can traverse in real-world space (see Figure 5D; e.g., Overman et al., 1996).

A dual-solution version of the radial arm maze may also be used to gauge the relative use of place and response learning strategies in human subjects (Figure 5C). In the dual-solution radial maze (Iaria et al., 2003), participants navigate a virtual maze and retrieve hidden reward objects by traveling to the ends of some arms. Given that there are numerous spatial cues in the distal virtual environment, including mountains and trees, the participants may use the spatial cues to determine which arms contain reward objects. However, the participant may also use an egocentric response strategy by learning the sequence of turns leading to the correct arms. To determine which strategy the participants employed, a probe test can be conducted in which walls surround the maze and prevent the subject from using the distal spatial cues. Thus, more errors during the probe test would suggest that the subjects had been using a place learning strategy, whereas few errors would suggest subjects had been using a response learning strategy. Also, as one of the advantages of performing experiments with human subjects, participants may also be debriefed and asked to report how they solved the task. From these responses, investigators can determine whether participants had used a place or response learning strategy.

### Morris Water Maze

**Figure 6:**
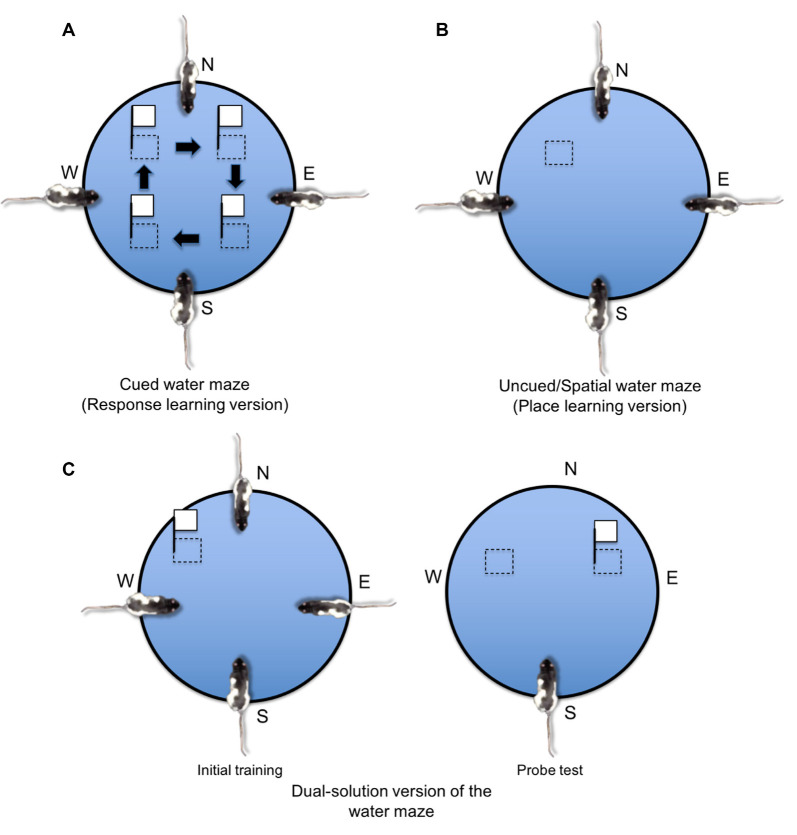
Place and response learning in the water maze.** (A)** In the cued water maze task, the platform is either visible (i.e., made of a conspicuous opaque material and/or is located above the water surface) or is cued with a proximal object (e.g., a white flag attached to the platform). Throughout training, the platform is rotated to different quadrants of the maze, and the animal must learn to associate the visible stimulus (S) with swimming approach behavior (R). **(B)** In the uncued spatial water maze task, the platform remains in a consistent spatial location and is visibly hidden below the water surface (the platform may consist of transparent material or the water may be infused with an opaque dye). Therefore, the rat must use extra-maze spatial cues to learn the location of the hidden platform. **(C)** In the dual-solution version of the water maze, a visibly cued platform remains in the same spatial location across initial training. During a later probe test, the cue is moved to a different maze quadrant. If the rat continues to swim to the same spatial location, the rat is labeled a place learner. If the rat approaches the visible cue, the rat is labeled a response learner.

Aside from the radial arm maze, place and response learning may also be investigated using the Morris water maze (see Figure 6; McDonald and White, 1994; Devan and White, 1999; Devan et al., 1999; Lee et al., 2008, 2014). In the standard place learning version of the Morris water maze (Figure 6B; Morris, 1984), rats are released into a circular pool of water from different starting positions and must rely on the allocentric spatial objects in the maze environment to learn the spatial location of an invisible escape platform. In the response learning or “cued” version (Figure 6A), the escape platform is visibly cued so that the animal may acquire an S-R association allowing the cued platform (S) to evoke approach behavior (R). The cued platform also moves to different spatial locations throughout training, making a spatial learning strategy unreliable. In a dual-solution version (Figure 6C), a cued platform remains in the same spatial location across training, allowing animals to acquire either a spatial learning strategy (i.e., go to the same spatial location) or a response learning strategy (i.e., go to the cued platform). What learning strategy the animal employed may be assessed using a probe trial in which the cued platform is moved to a new spatial location. If the rat continues to swim to the original spatial location, the rat is believed to have acquired a place learning strategy, whereas if the rat follows the cued platform to the new spatial location, the rat is believed to have acquired a response learning strategy.

### Barnes Maze

Place and response learning have also been investigated using the Barnes maze (Harrison et al., 2006; Rueda-Orozco et al., 2008; Wahlstrom et al., 2018). The Barnes maze is a large circular platform with a series of holes lining the perimeter where one of these holes leads to a small escape compartment underneath the maze. In the standard place learning version, the animal may use distal cues to acquire the spatial location of the escape hole (Barnes, 1979). In the cued or response learning version, the escape hole is located in different spatial locations across training but is reliably marked by a proximal visual cue (Reiserer et al., 2007). The relative use of place and response learning may also be examined using a dual-solution version of the Barnes maze, in which the escape hole is located in a consistent spatial location and is also reliably cued using a proximal visual stimulus (Harrison et al., 2006). During a subsequent probe trial, all the holes are blocked, and the proximal stimulus is relocated to a different hole. Spending more time near the original spatial location is indicative of a place learning strategy, whereas spending more time near the proximal cue is indicative of a response learning strategy. The relative use of place and response learning may also be observed by analyzing strategy use during the standard place learning version of the Barnes maze (Harrison et al., 2006; Rueda-Orozco et al., 2008). If animals run directly to the escape hole, they are considered to be using a place learning strategy. However, if animals run to an arbitrary hole and serially explore each adjacent hole until finding the correct hole leading to the escape compartment, they are considered to be using a response learning strategy.

### Outcome Devaluation

Outcome devaluation procedures can be added to place and response learning maze tasks to test whether the rat’s maze running behavior is goal-directed or habitual. Response learning in the plus-maze has been traditionally regarded as a type of learned S-R *habit* to the extent that the turning behavior (R) presumably occurs automatically in response to environmental stimuli (S). This is contrasted with place learning which requires the animal to purposefully guide behavior toward the goal using a cognitive map. In the 1980s, investigators began using outcome devaluation as a test to determine whether a learned behavior is habitual (Adams and Dickinson, 1981a,b; Adams, 1982; Dickinson and Nicholas, 1983; Dickinson et al., 1983). Outcome devaluation may be achieved in different ways but is perhaps most commonly implemented by pairing the food reinforcement with a lithium chloride injection that causes illness. Later, if the animal continues to perform the learned behavior even though the reinforcer has been devalued, the behavior is considered habitual. On the other hand, if the animal no longer performs the behavior after devaluation, the behavior is considered goal-directed. Although outcome devaluation was originally employed in instrumental learning tasks (e.g., lever pressing), this procedure may also be employed in place and response learning tasks to determine whether the maze running behavior in each task is habitual or goal-directed. Research has confirmed that response learning in the plus-maze, in addition to response learning in other maze tasks, is relatively insensitive to outcome devaluation, suggesting that the learned behavior is habitual (Sage and Knowlton, 2000; Lin and Liao, 2003; De Leonibus et al., 2011; Smith et al., 2012; Smith and Graybiel, 2013; Kosaki et al., 2018). In contrast, place learning proves sensitive to outcome devaluation, providing evidence that place learning is goal-directed (Sage and Knowlton, 2000; Lin and Liao, 2003; De Leonibus et al., 2011; Kosaki et al., 2018).

### The Place and Response Learning Plus-Maze Tasks in Other Species

Although the majority of studies employing the place and response learning tasks have used rats—and to lesser extent mice and humans—these tasks have been adapted for use across a wide variety of species. The purpose of many of these experiments has been to gauge the cognitive mapping abilities or spontaneous use of different navigational strategies across a range of species representing different branches on the phylogenetic tree. Taken together, these findings across species may allow for inferences to be made regarding the evolution of spatial navigation (see Jacobs, 2003; Salas et al., 2003).

Plus-maze versions of the place and response learning tasks—including the dual-solution task—have been employed to examine learning and memory in chickens (Brookshire et al., 1961), terrestrial toads (Daneri et al., 2011), horses (Parker et al., 2009), salamanders (Kundey et al., 2016), and turtles (López et al., 2000; Rodríguez et al., 2002; see Figure 4E). In addition to studies in terrestrial and amphibious animals, water plus-maze versions of the place and response learning tasks have been readily employed to study memory in a variety of aquatic animals, including sharks (Fuss et al., 2014a,b), freshwater stingrays (Schluessel and Bleckmann, 2005), cuttlefish (Alves et al., 2007), crayfish (Tierney and Andrews, 2013), and goldfish (Rodríguez et al., 1994, 2002; Salas et al., 1996a,b; Romaguera and Mattioli, 2008; McAroe et al., 2016). Aside from examining normal learning and memory abilities, some studies have used lesion techniques to examine the neural substrates of place learning in these animals. Lesions delivered to certain areas of the telencephalon believed to be homologous to the mammalian hippocampus, produce deficits in place learning, but not response learning, in sharks (Fuss et al., 2014a,b), goldfish (Salas et al., 1996a,b; Rodríguez et al., 2002; Romaguera and Mattioli, 2008), and turtles (Rodríguez et al., 2002). These findings demonstrate a similar role for the hippocampal formation in performing place learning functions across different species, suggesting that the ontogeny of hippocampal spatial memory processing may have an early evolutionary origin.

## Neural Mechanisms of Place and Response Learning: Evidence for Multiple Memory Systems

As noted above, the use of the place and response learning plus-maze tasks began to taper off following the resolution of the place vs. response controversy in the 1950s. However, these maze tasks were then revived decades later when investigators began exploring the neurobiology of learning and memory. In particular, place and response learning tasks became quite useful in research on multiple memory systems. The multiple memory systems hypothesis suggests that different types of memory are processed by different parts of the brain. This hypothesis has by now received extensive experimental support (for historical reviews, see Squire, 2004; White et al., 2013), and much of the evidence has come from the use of various place and response learning tasks. Importantly, while early research on memory systems suggested that memory systems operate independently and in parallel, there is also much evidence that memory systems can interact with each other cooperatively or competitively (Poldrack and Packard, 2003; Hartley and Burgess, 2005).

Place and response learning tasks have a major advantage in research on memory systems because they provide an overlapping experimental framework for examining double dissociations. When using the place and response learning tasks, a double dissociation may be demonstrated when damage to brain region A impairs place learning, but not response learning, whereas damage to brain region B impairs response learning, but not place learning. The major benefit to using the place and response learning tasks to demonstrate double dissociations is that these tasks involve similar motivational, sensory, and motoric processes, whereas the principal difference between the tasks is the type of memory required to solve each task. Therefore, if damage to brain region A disrupts acquisition in the place learning task, but not the response learning task, the effect may be attributed to an impairment in the type of memory underlying place learning rather than to an impairment in some non-mnemonic process. In contrast, if damage to brain region A impaired both place and response learning, it would be difficult to rule out the possibility that brain region A mediates a non-mnemonic process shared across both tasks. The present section reviews evidence that place and response learning are mediated by anatomically distinct neural systems, thus providing strong experimental support for the existence of multiple memory systems in the mammalian brain.

### Brain Regions

#### Hippocampus and DLS

The two chief brain structures that have been implicated in place and response learning in the plus-maze are the hippocampus and DLS. The differential mnemonic functions of the hippocampus and DLS in mediating cognitive spatial and S-R habit memory, respectively, were originally demonstrated using win-shift and win-stay versions of the eight-arm radial maze, as well as place and response learning versions of the Morris water maze (Packard et al., 1989; Packard and McGaugh, 1992; Packard et al., 1992; Devan and White, 1999; Devan et al., 1999; McDonald and White, 2013). Later research employed the classic Tolman and Blodgett plus-maze tasks to examine potential differences in the mnemonic functions of the hippocampus and DLS. In the dual-solution plus-maze task, temporary inactivation of the hippocampus leads to the predominant use of a response learning strategy, whereas inactivation of the DLS leads to the use of a place learning strategy (Packard and McGaugh, 1996; Ramos and Vaquero, 2000; Ramos, 2002; Middei et al., 2004a; Yin and Knowlton, 2004; Espina-Marchant et al., 2009; Schumacher et al., 2011; Jacobson et al., 2012; but see also Middei et al., 2004b). Also, intra-ventricular infusion of beta-amyloid protein, which is associated with Alzheimer’s disease and hippocampal memory deficits, leads to greater use of response learning over place learning in the dual-solution version of the task (Ammassari-Teule et al., 2002), and similar observations have been made in a transgenic mouse model of Alzheimer’s disease (Middei et al., 2004a, 2006).

Also, reversible or irreversible lesion of the hippocampus impairs acquisition in the place learning plus-maze task, but not in the response learning plus-maze task (Oliveira et al., 1997; Ramos, 2002; Schroeder et al., 2002; Chang and Gold, 2003a; Compton, 2004; Boucard et al., 2009; Jacobson et al., 2012). In fact, consistent with a competitive interaction between memory systems, sometimes inactivation of the hippocampus is associated with enhanced acquisition in the response learning plus-maze (Schroeder et al., 2002; Chang and Gold, 2003a; Compton, 2004). In contrast, reversible or irreversible lesion of the DLS impairs acquisition in the single-solution response learning plus-maze, but not in the place learning version of the task (Thompson et al., 1980; Chang and Gold, 2004; Compton, 2004; Asem and Holland, 2015; Gornicka-Pawlak et al., 2015).

The extensive research linking the hippocampus and DLS to place and response learning has strengthened the general view that these brain regions serve as the principal nodes of distinct memory systems (Squire, 2004; White et al., 2013). According to this view, the hippocampus is the central neural structure of a memory system mediating cognitive spatial learning and memory processes (O’Keefe and Nadel, 1978). In contrast, the DLS is the central neural structure of an S-R memory system responsible for linking sensory stimuli (S) in the learning environment with behavioral responses (R; for reviews, see Packard, 2009a; Goodman and Packard, 2016b).

#### Dorsomedial Striatum

The dorsal striatum is functionally heterogeneous. Whereas the DLS mediates S-R habit memory, the DMS mediates cognitive memory mechanisms akin to the hippocampus (for review, see Devan et al., 2011). Consistent with a role in spatial learning, DMS lesions impair acquisition in a variety of hippocampus-dependent spatial memory tasks, including place learning versions of the radial maze (Devan, 1997) and Morris water maze (Devan and White, 1999; Devan et al., 1999; Lee et al., 2014). In the dual-solution plus-maze, pre-training DMS lesions impair the use of a place learning strategy and lead to greater use of a response learning strategy (Yin and Knowlton, 2004). The role of the DMS in acquiring/expressing a place learning strategy may be partially attributed to dopaminergic mechanisms (Lex et al., 2011) and synaptic plasticity in the DMS (Hawes et al., 2015).

The DMS has also been critically implicated in reversal learning tasks. In a “response” reversal-learning task, rats are initially trained in a response learning version of the plus-maze to make a consistent body-turn response (e.g., turn left) and are subsequently given reversal training in which the opposite body turn (e.g., a right turn) is reinforced. In the “place” reversal task, rats are first trained in the place learning version of the plus-maze, in which a consistent spatial location (i.e., East arm) is reinforced, before receiving reversal training in which the opposite spatial location (e.g., West arm) is reinforced. Pre-training reversible or irreversible DMS lesions impair both place and response reversal learning (Pisa and Cyr, 1990; Ragozzino et al., 2002a; Ragozzino and Choi, 2004). Likewise, DMS lesions disrupt switching from a response learning strategy to a cue-guided strategy and vice versa (Ragozzino et al., 2002b). Glutamatergic and cholinergic mechanisms in the DMS may be required for reversal learning in these plus-maze tasks (Ragozzino et al., 2002b; Ragozzino, 2003; Palencia and Ragozzino, 2004, 2006; Ragozzino and Choi, 2004; Tzavos et al., 2004; McCool et al., 2008; Watson and Stanton, 2009; Ragozzino et al., 2009; Baker and Ragozzino, 2014).

Finally, similar to its well-established role in instrumental lever pressing (Yin et al., 2005a,b), the DMS has also been implicated in goal-directed responding in the dual-solution plus-maze task. In one experiment, mice received extensive training in the dual-solution task, so that they predominantly expressed a response learning strategy during the probe trial. The food reinforcer was subsequently devalued by pairing the reinforcer with a nausea-inducing lithium chloride injection (De Leonibus et al., 2011). Despite devaluation, the animals continued to seek the food reward, indicating S-R/habitual responding. However, when released from the opposite start arm, mice that had received reinforcer devaluation decreased the use of a response learning strategy, suggesting that devaluation may only influence response learning when the animal is released from a different starting position. In contrast, a response learning strategy was preserved in animals given DMS lesions, despite devaluation (De Leonibus et al., 2011). In sum, considering that the DMS receives input from hippocampal and amygdala circuitry (Groenewegen et al., 1987), the DMS is well-positioned to integrate spatial and motivational information to help generate appropriate behavioral output in maze learning situations. This may include both: (1) assisting in the development and execution of a place learning strategy; and (2) switching between strategies when a previously learned strategy no longer leads to a valued outcome.

#### Amygdala

Another brain region implicated in place and response learning is the basolateral complex of the amygdala (BLA). Although the BLA is not critically needed for the acquisition of place or response learning, this brain region may still be involved to the extent that it mediates the emotional modulation of memory in these tasks (for reviews, see Packard, 2009b; Packard and Goodman, 2012; Schwabe, 2013; Goodman et al., 2017a; Packard et al., 2018). As described above, stress/anxiety enhances the acquisition of response learning and impairs the acquisition of place learning. Also, stress/anxiety leads to the greater relative use of a response learning strategy in the dual-solution plus-maze. The BLA has been critically implicated in each of these effects. Intra-BLA administration of anxiogenic drugs is sufficient to enhance response learning, impair place learning, and lead to greater use of a response learning strategy in the dual-solution plus-maze (Packard and Wingard, 2004; Elliott and Packard, 2008; Wingard and Packard, 2008). Also, the enhancement of response learning produced by exposure to predator odor or systemic administration of anxiogenic drugs is blocked by neural inactivation of the BLA (Elliott and Packard, 2008; Packard and Gabriele, 2009; Leong and Packard, 2014). Likewise, enhancement of response learning produced by exposure to a fear-conditioned stimulus (i.e., tone previously paired with shock) is blocked following intra-BLA administration of the β-adrenergic receptor antagonist propranolol (Goode et al., 2016).

#### Medial Prefrontal Cortex

The medial prefrontal cortex has been implicated in a variety of cognitive executive functions and in particular has a critical role in behavioral flexibility (Ragozzino et al., 1999a,b; Block et al., 2007; Ragozzino, 2007). Behavioral flexibility may be defined generally as the ability to adapt one’s behavior to meet evolving task demands. This includes strategy switching, and indeed the medial prefrontal cortex has been implicated in switching from a place learning strategy to a response learning strategy, and* vice versa* (Ragozzino et al., 1999a,b; Rich and Shapiro, 2009). The infralimbic region by itself is required for the expression of response learning in the conditional T-maze task (Smith et al., 2012; Smith and Graybiel, 2013). For instance, optogenetic disruption of infralimbic activity disrupts retrieval of response learning in the conditional T-maze; however, after a new turning response has formed, inhibiting the infralimbic region a second time disrupts expression of this new turning response and restores expression of the old turning response (Smith et al., 2012).

Whereas the DLS, hippocampus, amygdala, and medial prefrontal cortex constitute the major brain regions popularly associated with place and response learning, there is some evidence that other brain regions are implicated in these tasks, including additional striatal and cortical brain areas (McDaniel et al., 1995; Cahill and Baxter, 2001; Noblejas and Poremba, 2003; Wang et al., 2011; Machado et al., 2014). These brain regions may cooperate within neural circuits to mediate the cognitive or S-R habit memory mechanisms underlying successful memory performance and strategy use in the place and response learning tasks.

### Neurotransmitter Systems

#### Glutamate

Glutamate serves as the major excitatory neurotransmitter in the brain and plays profound roles in synaptic plasticity and memory function, including the mnemonic processes underlying place and response learning. In a dual-solution plus-maze task, pre-training systemic administration of MK-801, an antagonist of the glutamate-sensitive NMDA receptor, does not impair initial acquisition but decreases the use of a place learning strategy and increases the use of a response learning strategy during a subsequent probe trial (Mackes and Willner, 2006). In another study, direct post-training infusions of glutamate into the hippocampus or DLS also influenced the relative use of place and response learning in the dual-solution plus-maze. As mentioned previously, control animals typically express a place learning strategy after limited training but then shift to the use of a response learning strategy following extensive training in the appetitive plus-maze. However, post-training infusion of glutamate directly into the hippocampus during initial acquisition of the dual-solution task is associated with the use of a place learning strategy even after extensive training, suggesting that intra-hippocampal glutamate blocks the shift to response learning (Packard, 1999). In contrast, post-training intra-DLS infusion of glutamate during initial acquisition in this task leads to greater use of a response learning strategy during the probe test even after only limited training, suggesting that intra-DLS glutamate accelerates the shift to response learning (Packard, 1999). Also, pre-training or post-training intra-DLS administration of the NMDA receptor antagonist AP5 impairs acquisition/consolidation of memory in the response learning version of the plus-maze (Palencia and Ragozzino, 2005; Leong and Packard, 2013). It is also worth highlighting that investigators have demonstrated similar roles for the hippocampus and DLS glutamatergic systems in place and response learning versions of the Morris water maze (Packard and Teather, 1997, 1999; Farina and Commins, 2020).

#### Dopamine

Extensive evidence has indicated a selective role for dopamine in the hippocampus and DLS in place and response learning, respectively (e.g., Packard and White, 1991; Packard and McGaugh, 1994; Packard et al., 1994; Packard and Teather, 1998; Legault et al., 2006). In one experiment using the response learning version of the plus-maze, systemic administration of either the dopamine D1 receptor antagonist SCH23390 or the dopamine D2 receptor antagonist eticlopride impaired acquisition of response learning (Daniel et al., 2006). In contrast, in a dual-solution plus-maze, catecholamine depletion in the hippocampus led to the predominant use of a response learning strategy (Roschlau and Hauber, 2017).

Electrophysiological evidence also indicates a role for DLS dopamine in response learning during memory performance in the T-maze. In one study (Lemaire et al., 2012), animals received unilateral dopamine depletion in the DLS. This dopamine depletion was associated with increased oscillations in local field potentials in the DLS during conditional T-maze performance, but only following extensive training in the task (Lemaire et al., 2012). In another study (Eddy et al., 2013), investigators found that wheel-running exercise enhanced acquisition in a tactile/visual version of the conditional T-maze task, and this partially depended on dopaminergic mechanisms. That is, intra-DLS infusion of the D1 receptor antagonist SCH23390 enhanced acquisition in the conditional T-maze task for the non-exercising rats but had no effect in the exercising rats. On the other hand, intra-DLS infusion of the D2 receptor antagonist eticlopride impaired T-maze acquisition for the exercising rats but did not affect the non-exercising rats. Thus, the mnemonic benefit of exercise in this task may depend on the downregulation of D1 receptor activity and upregulation of D2 activity in the DLS (Eddy et al., 2013).

In contrast to the DLS, the DMS as discussed above mediates the acquisition of a place learning strategy in the dual-solution plus-maze. Some evidence indicates that the role of the DMS in this kind of learning may partially depend on the dopaminergic system. That is, dopamine depletion in the DMS leads to the preferential use of a response learning strategy over a place learning strategy in the dual-solution plus-maze (Lex et al., 2011). In contrast, dopaminergic neurons in the ventral tegmental area and substantia nigra also play a role in the relative use of place and response learning, in that deleting NMDA receptors from these dopaminergic neurons impairs the use of a response learning strategy in the dual-solution plus-maze (Wang et al., 2011). However, another study indicated that daily exposure to atrazine for 1 year, which damages the striatonigral dopamine system, did not influence the relative use of place and response learning in the dual-solution task (Bardullas et al., 2013).

#### Acetylcholine

Several microdialysis studies have indicated that cholinergic mechanisms may be critically involved in place and response learning in the plus-maze. In a dual-solution plus-maze task, acetylcholine release in the hippocampus rises early in training (i.e., when animals typically use a place learning strategy) and remains elevated throughout extended training (Chang and Gold, 2003b). On the other hand, acetylcholine release in the DLS rises steadily throughout training and only asymptotes following extensive training when animals begin to express response learning during the probe trials (Chang and Gold, 2003b). Also, measures of acetylcholine release both before and during dual-solution training indicate that rats using a response learning strategy on a subsequent probe trial had a higher ratio of intra-DLS acetylcholine release relative to intra-hippocampal acetylcholine (McIntyre et al., 2003). This study also observed that acetylcholine release in the hippocampus was much higher for animals displaying a place learning strategy relative to animals displaying a response learning strategy (McIntyre et al., 2003). Similar findings suggesting a role for hippocampal and DLS acetylcholine release in place and response learning, respectively, have also been obtained in a dual-solution version of a Y-maze task (Pych et al., 2005a). Also, rats that were given pyrithiamine-induced thiamine deficiency, which presumably mimics the mnemonic impairments observed in Wernicke-Korsakoff syndrome, display greater acetylcholine release in the striatum and greater use of response learning in a dual-solution plus-maze task, relative to control rats (Vetreno et al., 2008).

In addition to the dual-solution task, a role for acetylcholine has also been observed in the single-solution place and response learning tasks. Acetylcholine release in the striatum is higher when animals are trained in the response learning version of the plus-maze task relative to the place learning version of the task, whereas hippocampal acetylcholine release increases similarly when animals are trained in the place or response learning task (Pych et al., 2005b). Interestingly, hippocampal acetylcholine release decreases when the response learning task is conducted in a homogenous visual surround, suggesting that a role for the hippocampus in response learning may be prevented by reducing extra-maze visual cues (Pych et al., 2005b).

A role for acetylcholine in place and response learning has also been observed through the ablation of cholinergic neurons in different brain areas. Selective ablation of cholinergic neurons in the striatum impairs acquisition in a conditional T-maze task but does not impair place learning in the Morris water maze (Kitabatake et al., 2003), suggesting that striatal acetylcholine may selectively support response learning. One brain region implicated in place and response learning is the medial septum/vertical limb of the diagonal band (MS/VDB), a brain region that releases acetylcholine into the hippocampus. Although lesion of the MS/VDB disrupts strategy preference in the dual-solution plus-maze task, selective ablation of the cholinergic neurons in this brain region fails to influence place and response learning (Cahill and Baxter, 2001). This suggests that the role of the MS/VDB in strategy preference may be achieved through other neurotransmitter systems not involving acetylcholine. However, in contrast to this study, another experiment indicated that selective ablation of MS/VDB cholinergic neurons enhanced the use of a place learning strategy in the dual-solution Morris water maze (Jonasson et al., 2004). To complicate matters even further, in direct contrast with this study, another experiment indicated that toxic ablation of MS/VDB cholinergic neurons impaired acquisition in a place learning version of the Morris water maze and led to greater use of a response learning strategy over a place learning strategy in a dual-solution version of the task (Janis et al., 1998). The reason for the discrepancies among these studies remains undetermined.

Regarding what receptor subtypes may be implicated in the effects of acetylcholine on place and response learning, evidence points to the involvement of muscarinic acetylcholine receptors. Intra-hippocampal infusion of the muscarinic receptor antagonist scopolamine impairs acquisition of the place learning version of the plus-maze while sparing acquisition in the response learning version of the plus-maze (Soares et al., 2013). In contrast, intra-DLS infusion of scopolamine impairs acquisition in the response learning plus-maze while preserving acquisition in the place learning plus-maze task (Soares et al., 2013). In another study using the dual-solution version of the Morris water maze, a higher ratio of muscarinic receptor binding in the hippocampus relative to the DLS was associated with preference for a place learning strategy over a response learning strategy (Grissom et al., 2013). In this same study, investigators found that a higher ratio of muscarinic receptor binding in the amygdala relative to the hippocampus was associated with a response learning strategy in the dual-solution Morris water maze (Grissom et al., 2013). Finally, high levels of choline acetyltransferase—an enzyme involved in acetylcholine synthesis—has been associated with the preferential use of a spatial strategy over a response learning strategy in a dual-solution version of the Morris water maze (Hawley et al., 2015).

#### Cannabinoids

Accumulating evidence suggests that the endocannabinoid system plays an important role in DLS-dependent memory processes (for review, Goodman and Packard, 2015a). Studies suggest that either disrupting or enhancing the function of the endocannabinoid system may impair response learning. Systemic or intra-DLS infusions of CB1 receptor agonists or antagonists have been associated with impaired acquisition in the response learning plus-maze task (Gerdeman et al., 2006, 2007; Goodman and Packard, 2014). Also, intra-DLS administration of the cannabinoid receptor agonist anandamide or antagonist AM251 impairs response learning in the Barnes maze (Rueda-Orozco et al., 2008), and systemic or intra-DLS injection of a cannabinoid receptor agonist WIN 55212-2 impairs consolidation of response learning in the Morris water maze (Goodman and Packard, 2014).

In contrast to acute administrations of cannabinoid drugs, repeated cannabis use may be associated with *enhanced* response learning. A history of cannabis use in humans leads to the preferential use of a response learning strategy in the virtual radial arm maze (Bohbot et al., 2013). The impairing and enhancing effects of short-term and long-term cannabinoid exposure on response learning have also been observed in instrumental learning tasks in rodents (Hilário et al., 2007; Crombag et al., 2010; Nazzaro et al., 2012; Gremel et al., 2016). The observation that repeated cannabinoid exposure strengthens habitual response learning is consistent with the hypothesis that repeated drug use may shift the control of behavior from hippocampal cognitive goal-directed learning to DLS response learning, as a potential neural mechanism underlying cannabis addiction (Goodman and Packard, 2015a, 2016a).

#### Estrogen

The mnemonic effects of estrogen in the place and response learning tasks have attracted considerable attention. As mentioned above, whether female rats display place learning or response learning in the dual-solution plus-maze partially depends on the estrous cycle (Korol et al., 2004). During proestrus (i.e., when ovarian hormone levels are high), female rats preferentially employ a place learning strategy, whereas during estrous (i.e., when ovarian hormones are low) female rats display a response learning strategy (Korol et al., 2004). However, it has been suggested that the influence of estrogen on learning strategy in the dual-solution plus-maze may only occur during the early stages of acquisition. Once the task is well learned, cycling estrogen does not influence the ability of female rats to use a place or response learning strategy in the dual-solution plus-maze (Schmidt et al., 2009).

Aside from simply examining the effects of endogenous cycling estrogen, the influence of estrogen on place and response learning may also be demonstrated through estrogen replacement in ovariectomized female rats. Estrogen replacement through systemic administration of estrogen or selective ERα or ERβ agonists enhance acquisition in the place learning version of the plus-maze and impairs acquisition in the response learning version of the plus-maze (Korol and Kolo, 2002; Hussain et al., 2013; Pisani et al., 2015), and similar effects of estrogen have been observed in the place and response learning versions of the eight-arm radial maze (Davis et al., 2005) and open-field tower maze (Lipatova et al., 2014). Besides, estrogen replacement through the administration of botanical compounds containing estrogenic properties may also enhance acquisition in the place learning plus-maze and impair acquisition in the response learning plus-maze (Pisani et al., 2012; Neese et al., 2014). Likewise, in a conditional T-maze task, estrogen replacement in ovariectomized rats impairs initial acquisition, yet enhances extra-dimensional set-shifting (Lipatova et al., 2016). Interestingly, the enhancing effect of very low estrogen levels on response learning in both the dual-solution and single-solution plus-maze tasks (e.g., Korol and Kolo, 2002) is blocked in female rats with prior reproductive experience (Hussain et al., 2013).

The effects of estrogen in these tasks may be attributed to estrogen activity in regions associated with place and response learning, i.e., the hippocampus, DLS, and medial prefrontal cortex. Direct infusion of estradiol into the hippocampus or DLS of female ovariectomized rats selectively enhances acquisition of place learning or response learning, respectively, in the Y-maze (Zurkovsky et al., 2007). Also, the increased use of a place learning strategy in the plus-maze during proestrus may be blocked by intra-hippocampal inactivation with muscimol (McElroy and Korol, 2005). Similar roles for the hippocampus and DLS have also been demonstrated using *c*-Fos immunohistochemistry labeling. Systemic estradiol administration is associated with increased *c*-Fos expression in the dentate gyrus, DMS, and DLS following acquisition in a place learning version of the plus-maze (Pleil et al., 2011). In contrast, systemic estradiol administration is associated with *decreased* *c*-Fos expression in the dentate gyrus, DMS, and DLS following acquisition in the response learning version of the plus-maze (Pleil et al., 2011). Importantly, in control animals, acquisition in the response learning task was associated with greater *c*-Fos expression in the DLS, whereas this increase was blocked by estradiol administration (Pleil et al., 2011). Estradiol-induced decrease of *c*-Fos activity in the DLS may be one factor contributing to the impairment in response learning following estradiol administration. Aside from hippocampal and striatal regions, evidence also indicates a role for the medial prefrontal cortex in the effects of estrogen on place and response learning. Infusion of estradiol into the medial prefrontal cortex, but not the anterior cingulate cortex, biases female rats toward the use of a place learning strategy over a response learning strategy in the dual-solution plus-maze (Almey et al., 2014).

Some evidence has suggested that estrogen might interact with the dopamine system to influence place and response learning. Estrogen replacement in ovariectomized rats augments the impairing effect of systemic administration of D2 receptor antagonist eticlopride, but not the impairing effect of D1 receptor antagonist SCH 23390, on the acquisition of response learning in the plus-maze (Daniel et al., 2006). The preference for response learning in the dual-solution task during low levels of estrogen may be reversed into a place learning preference following administration of either D1 receptor antagonist SKF 83566 or D2 receptor antagonist raclopride (Quinlan et al., 2008). Moreover, the preference for place learning produced by high levels of estrogen may be eliminated following SKF 83566 or raclopride administration, such that these animals subsequently show no preference for either place or response learning (Quinlan et al., 2008). In another study conducted in the dual-solution plus-maze, the response learning bias in low estrogen animals was reversed into a place learning bias following intra-DLS administration of the D1 receptor antagonist SCH 23390, but not the D2 receptor antagonist raclopride (Quinlan et al., 2013). Conversely, the place learning bias in high estrogen animals was reversed into a response learning bias following intra-DLS SCH 23390, but not intra-DLS raclopride administration (Quinlan et al., 2013). Although intra-DLS raclopride did not reverse strategy preference, a moderate dose of the drug was sufficient to eliminate strategy preference in the high- and low-estrogen animals, producing a comparable number of place and response learners in these groups. Also observed in this study was that administration of SCH 23390 or raclopride into the nucleus accumbens had no notable effects on strategy preference in high- or low-estrogen animals. Thus, the influence of high or low estrogen levels on strategy preference in the dual-solution task may depend on dopamine receptor activation selectively in the DLS (Quinlan et al., 2013). In a similar study, systemic administration of apomorphine or amphetamine at doses that increase D2 autoreceptor activity reversed the place learning bias into a response learning bias in high-estrogen animals, whereas no effects of these drugs were observed in the low estrogen rats (Hussain et al., 2016a). Also, amphetamine administration was associated with higher intra-DLS dopamine release in high estrogen rats relative to low estrogen rats, but in this study, DLS dopamine release itself was not reliably associated with strategy preference (Hussain et al., 2016a).

Finally, in contrast to studies using rodents in the plus-maze, a study in human subjects using a virtual radial arm maze (Hussain et al., 2016b) found that healthy, naturally cycling women showed a preference for response learning both during the early follicular phase (i.e., when estrogen levels are low) and the ovulatory phase (i.e., when estrogen levels are high) of the menstrual cycle. In contrast, women showed a preference for place learning over response learning during the mid/late luteal phase of the menstrual cycle (i.e., when estrogen levels are decreasing and progesterone levels are high). Therefore, the relative use of place and response learning in women similarly depends on the menstrual cycle, but the effects differ from what has been observed in research with rodents. Both estrogen and progesterone may be required for the dominant use of place learning in women.

#### Other Neurotransmitter Systems and Metabolic Substrates

A variety of other neurotransmitter systems have been implicated in place and response learning, albeit not as extensively as the neurotransmitters described above. For instance, low doses of testosterone lead to greater use of a response learning strategy in the dual-solution plus-maze and Morris water maze tasks, whereas a higher dose of testosterone leads to the predominant use of a place learning strategy in the dual-solution Morris water maze (Spritzer et al., 2013). Also, mice lacking delta-opioid receptors display a delay in the acquisition of a place learning strategy in the dual-solution plus-maze but show enhanced acquisition in the response learning version of the plus-maze (Le Merrer et al., 2013). In another study, mice lacking GPR88 receptors were quicker to acquire a dual-solution plus-maze task and also began using a response learning strategy sooner, relative to wild-type mice (Meirsman et al., 2015). Later, when the same group of mice was given reversal training in the dual-solution task, the GPR88 knockout mice were quicker to acquire the reversal and displayed a place learning strategy in a subsequent probe test, whereas the wild-type mice displayed a response learning strategy (Meirsman et al., 2015).

Aside from neurotransmitters, place and response learning may also depend on metabolic substrates, such as glucose and lactate. Increasing striatal function through injections of glucose into the DLS impairs acquisition in a place learning version of the Y-maze (Pych et al., 2006), which is consistent with a competitive interaction between the hippocampus and DLS memory systems (Poldrack and Packard, 2003). However, the intra-DLS infusions of glucose were not sufficient to facilitate acquisition in the response learning version of the Y-maze (Pych et al., 2006). In another study, extracellular levels of glucose in the hippocampus were significantly higher when rats were trained in a place learning plus-maze task, relative to rats trained in the response learning plus-maze task or control animals that received no training (see Gold et al., 2013). A similar pattern was observed for extracellular levels of lactate in the hippocampus, whereby animals trained in the place learning task had higher hippocampal lactate levels relative to animals trained in the response learning task or animals that received no training (Newman et al., 2017). Lactate level also increased in the DLS during response learning, but not place learning; however, this was only observed when drinking water was used as the reinforcement, not food (Newman et al., 2017). Metabolic substrates, such as glucose and lactate, may provide the necessary energy for neurons in the hippocampus and DLS to meet the demands of the place and response learning tasks, respectively.

## Conclusion

### Summary

The 75 years that have transpired since the original conception of the place and response learning plus-maze tasks (Tolman et al., 1946a; Blodgett and McCutchan, 1947, 1948) have instantiated their remarkable utility for examining behavioral and neurobiological mechanisms of learning and memory. The place and response learning plus-maze tasks were originally designed to assess Hullian S-R and Tolmanian cognitive learning theories. Proponents of the S-R view hypothesized that response learning would be the dominant form of learning expressed in these tasks, whereas proponents of the cognitive view hypothesized that place learning would be dominant. The eventual conclusion drawn from these experiments, however, was that either place or response learning may prevail in a given learning situation and that the dominance of cognitive or S-R learning systems depends on a variety of experimental variables.

The findings from these original studies anticipated the contemporary, widely held view that there are different kinds of memory. The place and response learning tasks have been employed to further demonstrate that these parallel kinds of memory are also mediated by different parts of the brain, which is consistent with the multiple memory systems view of learning and memory. In particular, extensive evidence indicates that the kind of memory underlying place learning depends on hippocampal function, whereas the kind of memory underlying response learning depends on DLS function. Based on these findings, numerous investigators have concluded that the hippocampus may constitute a principal part of the neural system mediating Tolmanian cognitive learning and memory, whereas the DLS may mediate Hullian S-R learning and memory (White and McDonald, 2002; Squire, 2004; White et al., 2013). Recently, place and response learning tasks have also been utilized extensively to examine the neurotransmitter systems involved in cognitive and S-R memory processes, indicating important roles for glutamate, dopamine, acetylcholine, cannabinoids, and estrogen.

### The Future of Place and Response Learning

The current widespread use of the place and response learning tasks may be extended to examine other neural mechanisms of learning and memory, including the role of neural circuits in multiple memory systems. Brain regions implicated in place and response learning, such as the medial prefrontal cortex, hippocampus, and dorsal striatum among others, are interconnected and therefore potentially interact both competitively and cooperatively to influence place and response learning. Glutamatergic projections arising from the hippocampal system and medial prefrontal cortex innervate the medial region of the dorsal striatum, and evidence suggests that these cortical and subcortical projections to the DMS may be implicated in spatial memory (e.g., Devan and White, 1999; Baker and Ragozzino, 2014). Likewise, there is evidence that frontostriatal circuitry is implicated in S-R response learning (Horga et al., 2015). However, the neural circuits involved in tilting the dominance from one memory system to another (e.g., from hippocampus-dependent place learning to DLS-dependent response learning) have yet to be identified, representing an open area for future work.

Although extensive research has examined the neural systems involved in the initial acquisition, consolidation, and retrieval of memory, few studies have investigated the *extinction* of memory in the place and response learning tasks. Some evidence suggests that extinction of place and response learning may involve different learning and memory processes mediated by distinct neural systems (Goodman and Packard, 2015b, 2018; Goodman et al., 2016a,b, 2017b). Therefore, these findings suggest that, like initial acquisition, extinction of place and response learning may also involve the distinct contributions of multiple memory systems (for reviews, see Goodman and Packard, 2018, 2019).

Another future direction of memory research that may benefit greatly from the use of place and response learning tasks is the continued development of memory systems theory. While there is strong evidence supporting the existence of distinct memory systems in the brain, some inconsistencies in the research have led investigators to propose amendments to memory systems theory (Murray et al., 2017; Ferbinteanu, 2018). For instance, under certain training parameters, the hippocampus may be required for response learning, and the DLS may be required for place learning (Ferbinteanu, 2016, 2020), which is in stark contrast to the conventional view that the hippocampus mediates place learning and the DLS mediates response learning (White et al., 2013). To accommodate these inconsistent findings, it has been suggested that memory systems may operate as neural networks with the ability to adapt to changing environmental demands (Ferbinteanu, 2018). The ongoing use of the place and response learning tasks will likely aid in the continued revision of memory systems theory, thus further strengthening our understanding of how memory is organized in the brain.

Finally, studies using the place and response learning tasks may also be relevant for understanding the behavioral and neurobiological mechanisms of some human psychopathologies. An emerging hypothesis proposed by multiple investigators is that DLS-dependent memory processes may contribute to the habit-like behavioral symptoms of some human psychiatric disorders (Graybiel and Rauch, 2000; Goodman et al., 2012, 2014; Berner and Marsh, 2014; Gillan and Robbins, 2014; Corbit and Janak, 2016; Packard et al., 2018). In particular, the shift from recreational drug use to compulsive drug abuse may reflect a shift in control from hippocampus-dependent cognitive memory to DLS-dependent S-R habit memory (White, 1996; Everitt and Robbins, 2005, 2013; Schwabe et al., 2011; Goodman and Packard, 2015a, 2016a). Thus, the transition from cognitive place learning to habitual response learning in the plus-maze may serve as an experimental model for understanding the mechanisms underlying maladaptive habits in drug addiction, as well as habit-like symptoms in other human psychopathologies.

## Author Contributions

JG wrote the article.

## Conflict of Interest

The author declares that the research was conducted in the absence of any commercial or financial relationships that could be construed as a potential conflict of interest.

## Footnotes

^1^The use of the term “plus-maze” in the present review denotes mazes with four arms arranged in a cross (+) orientation in addition to mazes with three arms arranged in a “T” formation. Although the latter maze is usually referred to as a T-maze, the present review opts to use the term plus-maze to refer to both types of mazes. This is because when a T-maze is employed in studies of place and response learning, the maze is usually rotated 180° throughout the learning task and therefore serves the same function as a plus-maze.
